# *TP63* gain-of-function mutations cause premature ovarian insufficiency by inducing oocyte apoptosis

**DOI:** 10.1172/JCI162315

**Published:** 2023-03-01

**Authors:** Chengzi Huang, Simin Zhao, Yajuan Yang, Ting Guo, Hanni Ke, Xin Mi, Yingying Qin, Zi-Jiang Chen, Shidou Zhao

**Affiliations:** 1 Center for Reproductive Medicine and; 2 Key Laboratory of Reproductive Endocrinology of Ministry of Education, Shandong University, Jinan, Shandong, China.; 3 Shandong Key Laboratory of Reproductive Medicine, Jinan, Shandong, China.; 4 Shandong Provincial Clinical Research Center for Reproductive Health, Jinan, Shandong, China.; 5 Shandong Technology Innovation Center for Reproductive Health, Jinan, Shandong, China.; 6 National Research Center for Assisted Reproductive Technology and Reproductive Genetics, Shandong University, Jinan, Shandong, China.; 7 Research Unit of Gametogenesis and Health of ART-Offspring, Chinese Academy of Medical Sciences, Jinan, China.; 8 Shanghai Key Laboratory for Assisted Reproduction and Reproductive Genetics, Shanghai, China.; 9 Center for Reproductive Medicine, Ren Ji Hospital, School of Medicine, Shanghai Jiao Tong University, Shanghai, China.

**Keywords:** Genetics, Reproductive Biology, Apoptosis, Fertility, Genetic diseases

## Abstract

The transcription factor p63 guards genome integrity in the female germline, and its mutations have been reported in patients with premature ovarian insufficiency (POI). However, the precise contribution of the *TP63* gene to the pathogenesis of POI needs to be further determined. Here, in 1,030 Chinese patients with POI, we identified 6 heterozygous mutations of the *TP63* gene that impaired the C-terminal transactivation-inhibitory domain (TID) of the TAp63α protein and resulted in tetramer formation and constitutive activation of the mutant proteins. The mutant proteins induced cell apoptosis by increasing the expression of apoptosis-inducing factors in vitro. We next introduced a premature stop codon and selectively deleted the TID of TAp63α in mice and observed rapid depletion of the *p63*^+/*Δ*TID^ mouse oocytes through apoptosis after birth. Finally, to further verify the pathogenicity of the mutation p.R647C in the TID that was present in 3 patients, we generated *p63^+/R647C^* mice and also found accelerated oocyte loss, but to a lesser degree than in the *p63*^+/*Δ*TID^ mice. Together, these findings show that TID-related variants causing constitutive activation of TAp63α lead to POI by inducing oocyte apoptosis, which will facilitate the genetic diagnosis of POI in patients and provide a potential therapeutic target for extending female fertility.

## Graphical abstract

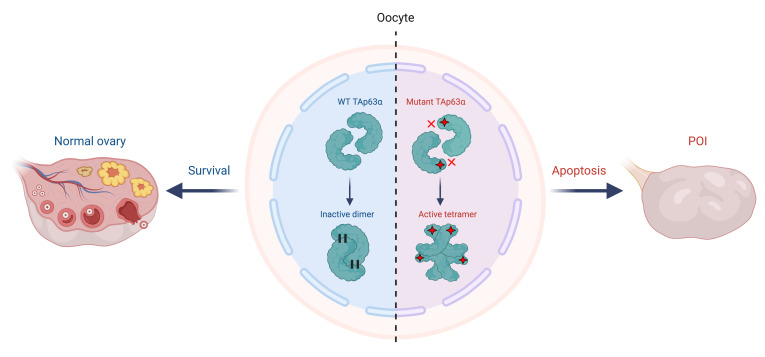

## Introduction

Premature ovarian insufficiency (POI) is characterized by the decline of ovary activity, with increasing follicle-stimulating hormone (FSH) levels (FSH >25 IU/L) and low estradiol (E_2_) levels before the age of 40 years (1). POI has a global prevalence of approximately 3.7% and dramatically decreases female reproductive capacity and impairs overall health (2). POI can either be isolated or part of a syndrome, and its etiology, including genetic defects, autoimmune disorders, infections, iatrogenic injuries, and environmental toxins, is highly heterogeneous (3). Substantial progress has been made in determining the genetic factors underlying POI. Chromosomal abnormalities, which have a prevalence of 10%–15% in patients with POI, together with gene mutations affecting oogenesis and folliculogenesis, account for a total of 20%–25% of POI cases (4). Recently, high-throughput sequencing, especially whole-exome sequencing (WES), has identified a variety of POI-causative genes, which emphasizes the crucial role of the meiosis and DNA repair pathways in ovarian development and function (5, 6). However, the etiologies of most POI cases remain unexplained.

The *p53* gene family member *p63* (*TP63* in humans) is transcribed by 2 different promoters, generating either the TAp63 isoforms containing the full-length transactivation domain (TAD) or the ΔNp63 isoforms without the TAD (7). In addition, because of alternative splicing, TAp63 and ΔNp63 usually possess different C-termini — referred to as α, β, and γ — to generate 6 isoforms (8). These different isoforms are expressed in specific tissues and execute distinct functions (9). For example, ΔNp63α is highly expressed in epithelial tissues and regulates their development and differentiation (9, 10), whereas TAp63α is specifically expressed in the oocytes of primordial follicles and plays a decisive role in oocyte survival after DNA damage (11–13). Under physiological conditions, TAp63α is maintained in an inactive dimeric state by the binding of its C-terminal transactivation-inhibitory domain (TID) with the N-terminal TAD (14–16). When DNA damage occurs, TAp63α is activated through sequential phosphorylation catalyzed by the kinases CHK2 and CK1, and this triggers the transition from an inactive dimer to an open and active tetramer (17, 18). Activated TAp63α induces apoptosis in oocytes by directly activating the transcription of its target genes, including *Puma*, *Noxa*, and *Bax* (19–22). Thus, TAp63α serves as a guardian of the female germline by eliminating damaged oocytes (23, 24).

In previous reports, heterozygous variants in the human *TP63* gene were mostly shown to impair epidermal development and to cause multiple organ malformations, including 5 syndromic and 2 nonsyndromic disorders (25). Interestingly, a relationship has been identified between C-terminal variations of the *TP63* gene and POI (26–29). The patients in these studies with *TP63* mutations presented with syndromic or isolated POI, and these variants were frameshift or nonsense mutations leading to truncated C-termini, which might disrupt the autoinhibitory state of TAp63α and induce uncontrolled oocyte death (30). However, the pathogenicity of these mutations has been largely speculative without convincing evidence. Furthermore, given the limited number of patients reported, the precise contribution, the genotype-phenotype correlation, and the clinical significance of POI-related *TP63* mutations remain largely unknown.

In this study, in addition to truncating mutations, we identified what we believe to be 4 novel missense mutations of *TP63* in a large database of 1,030 women with isolated POI and performed a comprehensive functional validation of the mutations using both in vitro and in vivo studies. We demonstrated that these gain-of-function mutations of *TP63* caused POI by inducing oocyte apoptosis and accounted for 0.78% (8 of 1,030) of the studied cases. Our results also suggest that different mutation types with distinct transactivation activities determine the rate of oocyte loss and phenotype variability. Here, we confirm that *TP63* is a POI-causative gene and provide strong evidence that its identification allows for early diagnosis and intervention for both female and male carriers of mutations of this gene.

## Results

### Variants of the TP63 gene were identified in patients with POI.

Through successive filtration steps of WES data from 1,030 unrelated individuals diagnosed with isolated POI, 9 heterozygous variants in *TP63* (NM_003722.5: p.S285N, p.T538A, p.T567I, p.Q568fs*3, p.R594*, p.R643Q, p.L646P, p.R647C, and p.R655Q) were identified in 11 patients with POI and were confirmed by Sanger sequencing (Table 1 and Supplemental Figure 1A; supplemental material available online with this article; https://doi.org/10.1172/JCI162315DS1). These patients presented with primary or secondary amenorrhea, and the age of amenorrhea ranged from 13 to 29 years. Detailed clinical characteristics are shown in Supplemental Table 1. Eight of the mutations were, to our knowledge, novel variants, while the mutation p.R594* was previously reported (28). Three unrelated individuals carried the same p.R647C mutation, which was identified in a patient with POI during the revision of our manuscript (31). The amino acid sites of all identified mutations were highly conserved across species, and these mutations were mostly predicted to be damaging (Table 1).

**Figure 1:**
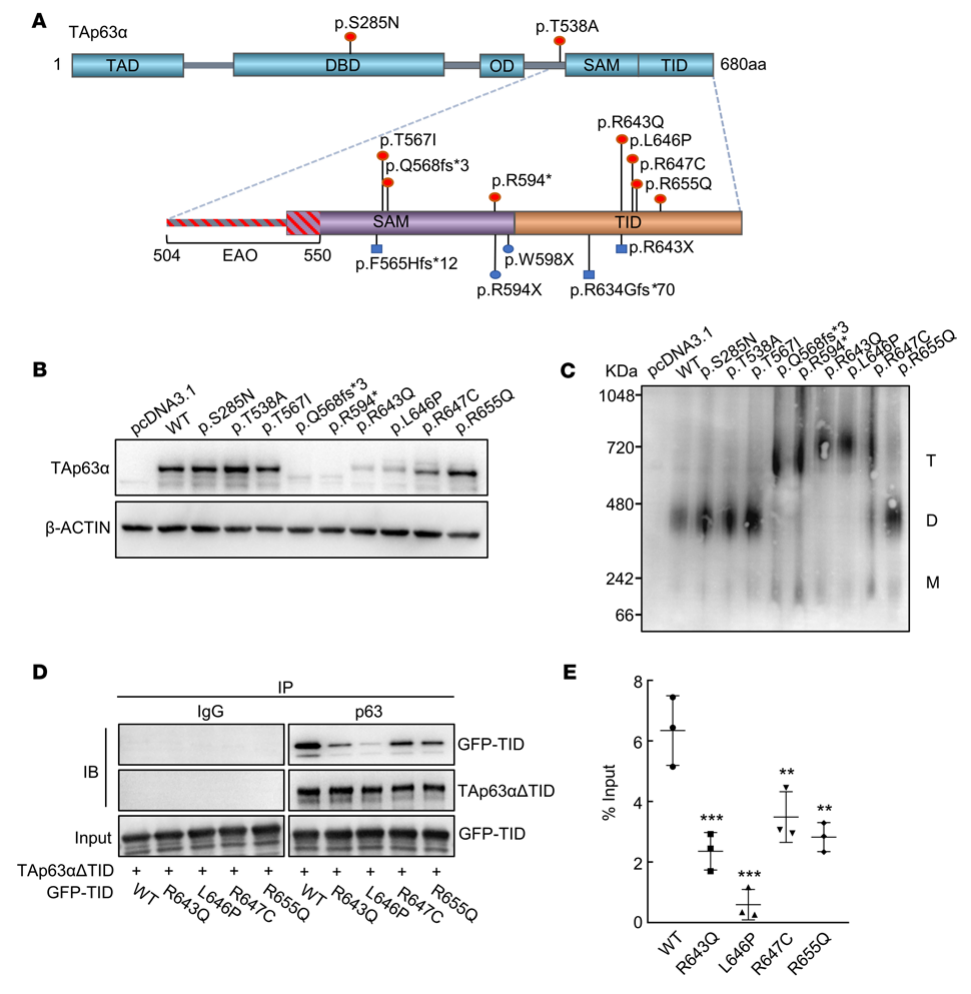
Analysis of human TAp63α-mutant pathogenicity. (**A**) Schematic diagram showing the 5 key domains of TAp63α: the TAD, the DBD, the oligomerization domain (OD), the SAM, and the TID. The positions of the variants identified in this study and reported in the literature are indicated in red and blue, respectively. Circles signify isolated POI; squares signify syndromic POI. The amino acid sequence of the EAO (504~550) is indicated. (**B**) After transfection with WT and mutant TAp63α plasmids in SAOS-2 cells, the intracellular protein level of TAp63α was detected by Western blotting. β-Actin was used as the loading control. (**C**) Oligomeric state analysis of WT and mutant TAp63α by BN-PAGE. The oligomeric conformation is indicated by T (tetramer), D (dimer), and M (monomer). In the protein samples of mutant TAp63α, no WT protein was present. (**D**) TAp63αΔTID and WT or mutant GFP-TID plasmids were cotransfected into HEK293 cells. Cells were harvested for co-IP assays and were immunoprecipitated with anti-p63 antibody, and then WT and mutant GFP-TID protein were detected by GFP antibody by Western blotting. IgG was used as the negative control. (**E**) Quantitative analysis of the amount of co-IP between TAp63αΔTID and WT or mutant GFP-TID. The immunoprecipitated GFP-TID was compared with the input. Data are presented as the mean ± SD of 3 independent experiments. **P < 0.01 and ***P < 0.001, by 1-way ANOVA followed by Dunnett’s test.

While the p.S285N and p.T567I mutations were in the DNA-binding domain (DBD) and the sterile α motif (SAM) domain, respectively, the p.T538A mutation was not located in any known functional domain. It is noteworthy that the 6 other mutations affected the C-terminal TID, which is essential for forming the inactive dimer (14, 15). Both the frameshift mutation p.Q568fs*3 and the nonsense mutation p.R594* fell within the SAM domain and truncated the TAp63α protein prior to the TID. Interestingly, these 2 mutations and 5 previously reported POI-related mutations were all heterozygous frameshift or nonsense mutations leading to the loss of all or part of the TID (Figure 1A) (26–29). In addition, the other 4 point mutations we identified (p.R643Q, p.L646P, p.R647C, and p.R655Q) were located in the conserved core sequence of the TID from R643 to R655 (RFTLRQTISFPPR), which is responsible for binding with the TAD (Supplemental Figure 2) (16). Furthermore, we confirmed that p.R643Q was paternally inherited (Supplemental Figure 1, B and C). Together, these results indicate that mutations impairing the TID might make a dominant contribution to POI pathogenesis.

### Mutant TAp63α proteins impair the stable oligomeric state.

To elucidate the effects of the mutations, we overexpressed the WT and mutant TAp63α proteins in human SAOS-2 cells, which is an osteosarcoma cell line without expression of TP53 or TP63 (32). We first measured the expression of TAp63α protein by Western blotting and found that WT and 4 mutant proteins (p.S285N, p.T538A, p.T567I, and p.R655Q) were highly expressed but that other mutant proteins were hardly detectable (p.Q568fs*3 and p.R594*) or were significantly reduced (p.R643Q, p.L646P, and p.R647C) (Figure 1B). Considering that active forms of TAp63α are difficult to detect because of their high turnover rate (14, 33), this result implies that some of these affected mutant proteins might be autoactivated.

To gain insight into the oligomeric state of these mutant proteins, we inhibited their proteasome-dependent degradation by treatment with MG132 and performed a blue native–PAGE (BN-PAGE) analysis. The results showed that all of the TID-related mutants (p.Q568fs*3, p.R594*, p.R643Q, p.L646P, p.R647C, and p.R655Q) formed tetramers, whereas the other mutants (p.S285N, p.T538A, and p.T567I) remained dimeric (Figure 1C), suggesting that only TID-related variants were spontaneously activated. However, the p.R655Q mutant was mainly dimeric and did not achieve a high tetramer/dimer ratio. Previous studies have demonstrated that the TID binds to the N-terminal TAD of TAp63α as an intramolecular mechanism to inhibit its transcriptional activity (14, 16). Thus, it was not surprising that the TID loss caused by p.Q568fs*3 and p.R594* led to the activation of TAp63α. We then speculated that the 4 point mutations located in the core sequence of the TID might disrupt the binding between the TAD and the TID, thus altering the dimeric conformation of the TAp63α protein. To test this hypothesis, we performed co-IP experiments by cotransfection of TAp63αΔTID, which possesses the TAD but without the TID, with plasmids containing the WT or mutant TID. Compared with the WT TID, the p.R643Q, p.L646P, p.R647C, and p.R655Q mutations significantly reduced the interaction between the TID mutants and theTAp63αΔTID (Figure 1, D and E). Collectively, these results suggest that TID-related variants disrupt the inactive conformation and generate constitutively active mutants.

### TAp63α mutants activate the apoptotic pathway.

**Figure 2:**
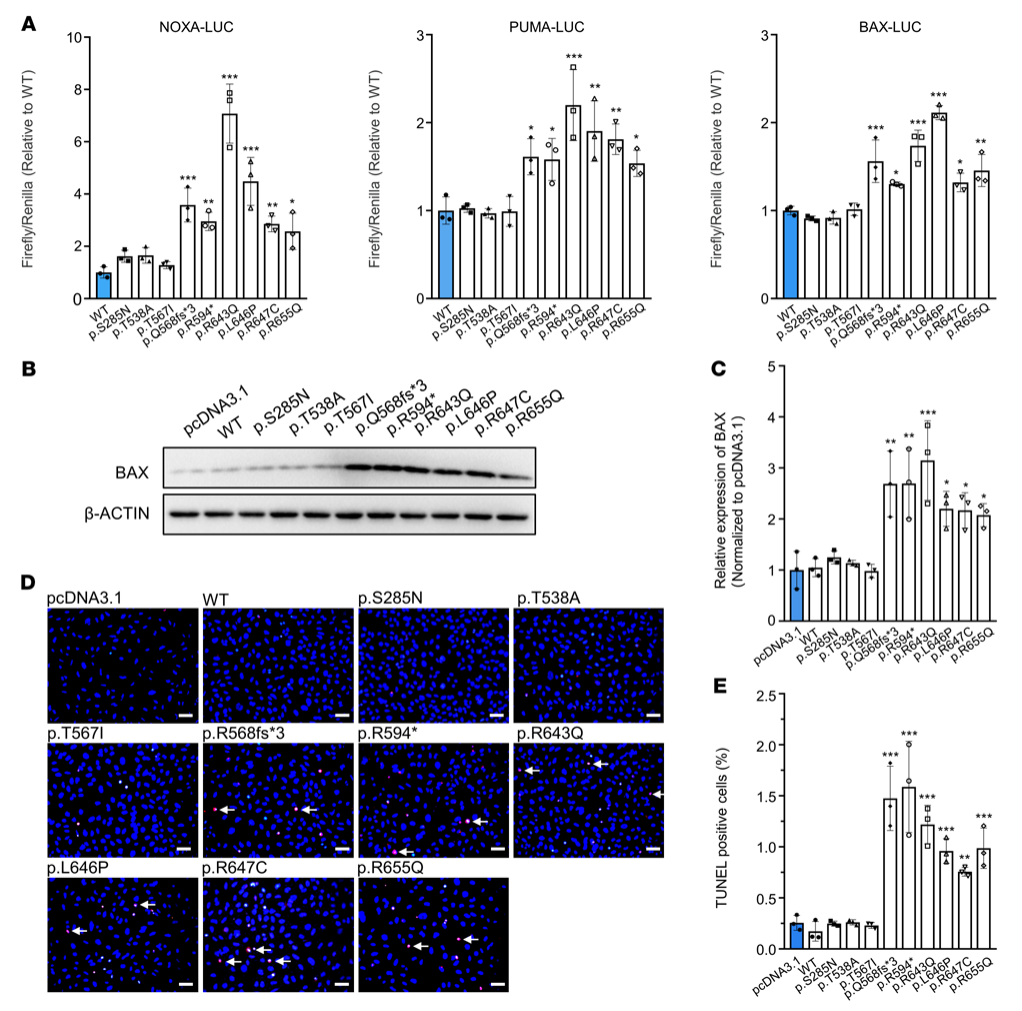
TAp63α mutants induce cell apoptosis. (**A**) Relative transcriptional activity of TAp63α mutants in SAOS-2 cells on *NOXA*, *PUMA*, and *BAX* promoters. The activity of the WT and mutant TAp63α is shown in blue and white, respectively. The activity of the WT group was set to 1. LUC, luciferase. (**B**) Western blot analysis of BAX expression after transfection with WT and mutant TAp63α plasmids in SAOS-2 cells. β-Actin was used as the loading control. (**C**) Quantification of BAX protein expression. (**D**) TUNEL staining of SAOS-2 cells after transfection with WT and mutant TAp63α plasmids. TUNEL-positive signals are indicated by arrows. Cell nuclei were counterstained with DAPI (blue). Scale bars: 50 μm. (**E**) Quantitative analysis of TUNEL-positive SAOS-2 cells after transfection. In panel **A**, **C** and **E**, data are presented as the mean ± SD of 3 independent experiments. *P < 0.05, **P < 0.01, and ***P < 0.001, by 1-way ANOVA followed by Dunnett’s test.

To further address the functional consequences of these mutations, we measured the transcriptional activity of the 9 mutants on the *PUMA*, *NOXA*, and *BAX* promoters using the luciferase reporter assay. Compared with WT TAp63α, the 6 TID-related mutants (p.Q568fs*3, p.R594*, p.R643Q, p.L646P, p.R647C, and p.R655Q) showed significantly increased transcriptional activity toward the 3 reporters (Figure 2A). We noticed that the transcriptional activity was inversely proportional to the detected protein levels of TAp63α, which was in agreement with a previous study (16). We also found that the 6 TID-related mutants significantly increased BAX protein expression (Figure 2, B and C). Consistent with this, the apoptosis assays also showed a significant increase in TUNEL-positive SAOS-2 cells overexpressing the TID-related mutants (Figure 2, D and E). These results demonstrate that mutations affecting the TID spontaneously activate TAp63α, increase the expression of its target genes, and induce apoptosis, presumably leading to premature exhaustion of oocytes and the occurrence of POI. Taken together, the 6 TID-related *TP63* mutations were evaluated to be pathogenic according to the guidelines of the American College of Medical Genetics and Genomics (34) (Table 1).

### p63^+/ΔTID^ mice exhibit rapid oocyte loss and a POI-like phenotype.

**Figure 3:**
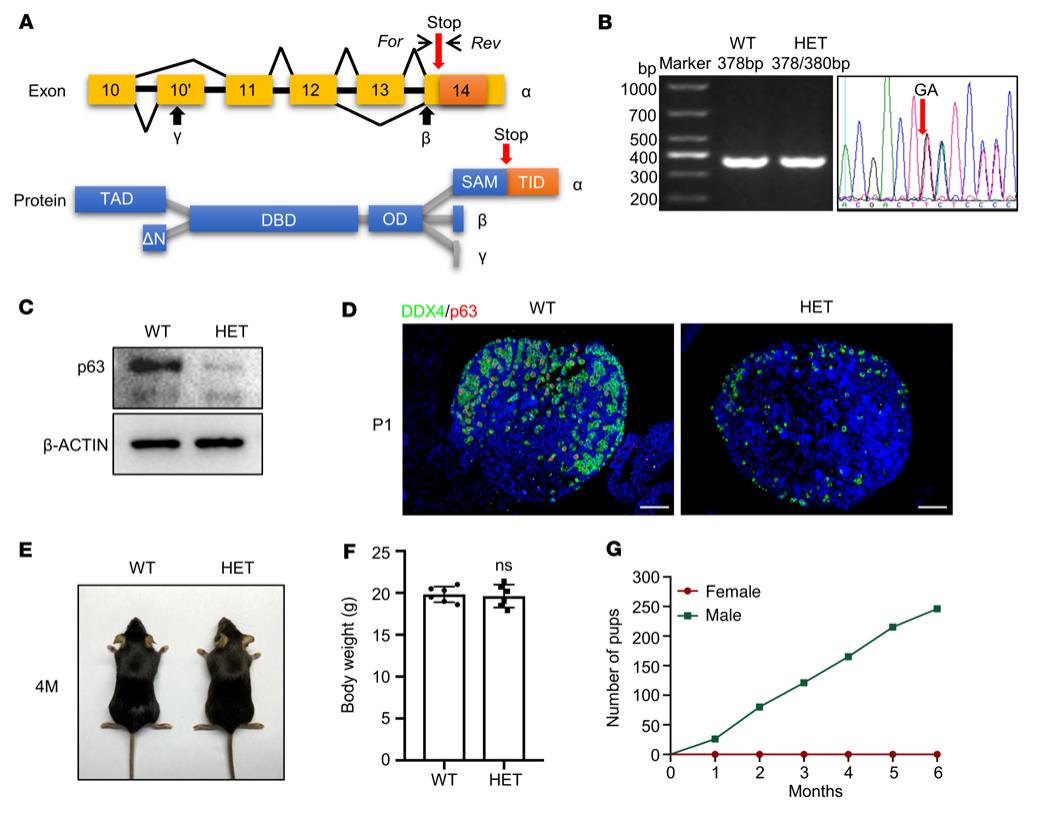
Generation and characterization of *p63^+/ΔTID^* mice. (**A**) Strategy for the generation of *p63*^+/ΔTID^ (referred to in the figures as HET) mice. The c.1828_1829insGA (NM_001127259.1) mutation was introduced into exon 14, leading to the formation of a stop codon and loss of the TID. The primers used for genotyping the WT and *p63*^+/ΔTID^ mice are shown. For, forward; Rev, reverse. (**B**) Agarose gel electrophoresis of the PCR products obtained from genomic DNA of WT and *p63*^+/ΔTID^ mice. Sanger sequencing confirmed the creation of the insert mutation. (**C**) Western blot analysis of p63 expression in protein extracts from P1 WT and *p63*^+/ΔTID^ mouse ovaries. β-Actin was used as the loading control. (**D**) IF staining for DDX4 (green) and p63 (red) in ovary sections from P1 WT and *p63*^+/ΔTID^ mice. Cell nuclei were counterstained with DAPI (blue). Scale bars: 50 μM. (**E**) Gross morphology of 4M WT and *p63*^+/ΔTID^ females. (**F**) No significant difference in body weights was observed between 4M WT and *p63*^+/ΔTID^ mice. *n* = 6 per group. An unpaired, 2-tailed Student’s *t* test was used for the comparison of the 2 groups. (**G**) Number of pups obtained by crossing *p63*^+/ΔTID^ males (green line) and *p63*^+/ΔTID^ females (red line) with WT mice for a period of 6 months. *n* = 6 per group.

To define the role of the p63 TID in POI pathogenesis in vivo, we specifically deleted the TID by inserting 2 nucleotides to introduce a stop codon prior to the TID in exon 14 of the *p63* gene in mice (Figure 3A). This strategy selectively altered the *p63*α isoform, while the *p63*β and *p63*γ transcripts remained unaffected (Figure 3A and Supplemental Table 2). The genotype of the *p63*^+/ΔTID^ mouse was verified by Sanger sequencing (Figure 3B), and p63 protein was detected at a very low level in the ovary of the P1 *p63*^+/ΔTID^ mouse by Western blotting (Figure 3C). Loss of p63 protein was also verified by immunofluorescence (IF) staining of the P1 *p63*^+/ΔTID^ mouse ovary in which significant oocyte loss was observed (Figure 3D). The *p63*^+/ΔTID^ mice were viable, and we observed no apparent abnormalities (Figure 3E). There were no significant differences in body weight between adult WT and *p63*^+/ΔTID^ mice (Figure 3F). Furthermore, H&E and IF staining showed no obvious abnormalities in skin structures, cell proliferation, or differentiation of the dorsal epidermis in newborn *p63*^+/ΔTID^ mice (Supplemental Figure 3). However, the *p63*^+/ΔTID^ females were infertile, whereas the *p63*^+/ΔTID^ males were fertile (Figure 3G).

**Figure 4:**
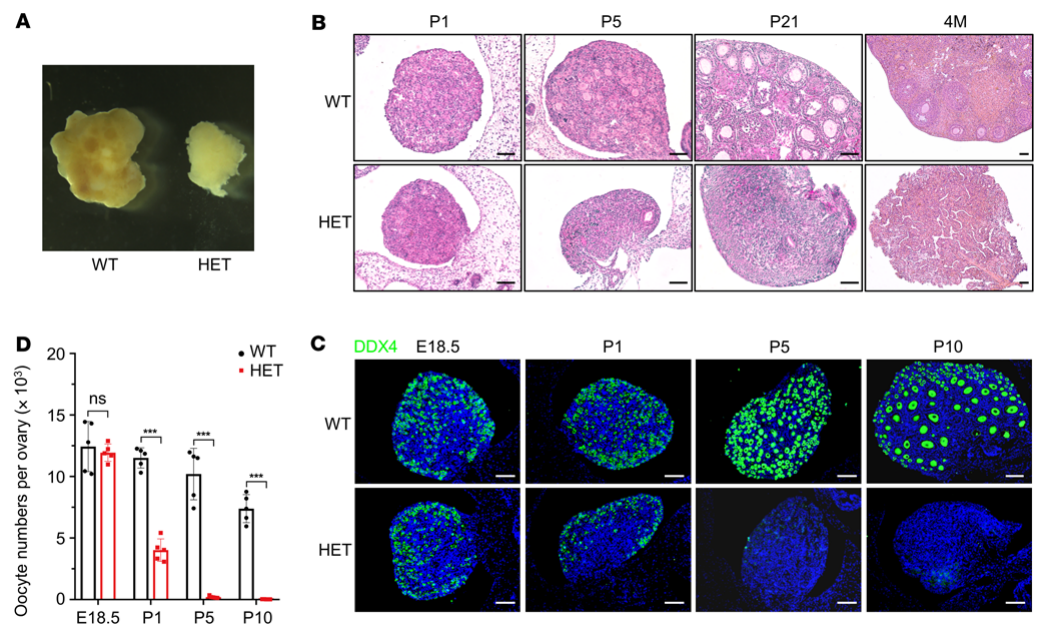
*p63^+/ΔTID^* mice show rapid oocyte loss. (**A**) The size of ovaries from 4M WT and *p63*^+/ΔTID^ females. (**B**) H&E staining of ovary sections from WT and *p63*^+/ΔTID^ mice at P1, P5, P21, and 4M. Scale bars: 50 μM. (**C**) IF staining for DDX4 (green) in ovary sections from E18.5, P1, P5, and P10 WT and *p63*^+/ΔTID^ mice. Cell nuclei were counterstained with DAPI (blue). Scale bars: 50 μM. (**D**) Quantitative analysis of DDX4-expressing oocyte numbers per ovary in each group. *n* = 5. Data are presented as the mean ± SD. ***P < 0.001, by unpaired, 2-tailed Student’s *t* test.

We carried out a detailed analysis of the ovarian tissues to reveal the infertility phenotype of *p63*^+/ΔTID^ females. The ovary size of 4-month-old (4M) *p63*^+/ΔTID^ mice was markedly reduced (Figure 4A). Ovarian sections with HE staining showed that follicles were substantially reduced at P1, barely visible at P5, and completely absent at P21 and 4M (Figure 4B). To determine the timing of oocyte loss, we performed IF staining for DDX4, a germ cell marker, in ovaries from WT and *p63*^+/ΔTID^ mice at E18.5, P1, P5, and P10 (Figure 4C). Although we found no significant differences in oocyte numbers between E18.5 *p63*^+/ΔTID^ embryos and their littermate controls, the oocyte numbers in P1 *p63*^+/ΔTID^ mice were decreased to approximately 40% of those in WT mice (Figure 4D). Few oocytes in *p63*^+/ΔTID^ mice survived at P5, and they completely disappeared by P10 (Figure 4D). In addition, we detected elevated FSH and decreased estradiol (E_2_) levels in the serum of 2M *p63*^+/ΔTID^ mice (Supplemental Figure 4A). These results suggest that expression of the mutant p63 without the TID resulted in rapid depletion of oocytes and loss of fertility, similar to the phenotype of human POI.

To further determine the extra-ovarian effects of TID deletion, we detected *p63* mRNA expression in the skin, ovary, hypothalamus, and pituitary of WT and *p63*^+/ΔTID^ mice (Supplemental Figure 4, B–D). The results showed that *p63*α was not detected in the hypothalamus or pituitary, suggesting that the TID deletion would have no effect on these 2 tissues. We observed elevated mRNA expression of the common gonadotropin α subunit (*Cga*), follicle-stimulating hormone β subunit (*Fshb*), and luteinizing hormone β subunit (*Lhb*) in the *p63*^+/ΔTID^ pituitary, while gonadotropin-releasing hormone (*Gnrh)* mRNA expression in the hypothalamus was not significantly changed (Supplemental Figure 4, E and F). Taken together, these results suggested that both the hypothalamus and pituitary functioned normally in *p63*^+/ΔTID^ mice and that the increased expression of *Fsh* and *Lh* in the pituitary might be a compensation for the decreased ovarian function.

Considering that TAp63 acts as a master transcriptional regulator of lipid and glucose metabolism (35), we determined the metabolic profiles of the *p63*^+/ΔTID^ mice. There were no obvious differences in body weight or liver lipid deposition in 12M *p63*^+/ΔTID^ mice compared with WT mice (Supplemental Figure 5, A–C), and there were no obvious changes in fasting glucose levels, glucose tolerance, or serum total cholesterol or triglyceride levels in 2M *p63*^+/ΔTID^ mice compared with WT mice (Supplemental Figure 5, D–H). These results indicated that TID deletion did not obviously influence metabolism in the mice.

### p63^+/ΔTID^ oocytes undergo uncontrolled apoptosis.

**Figure 5:**
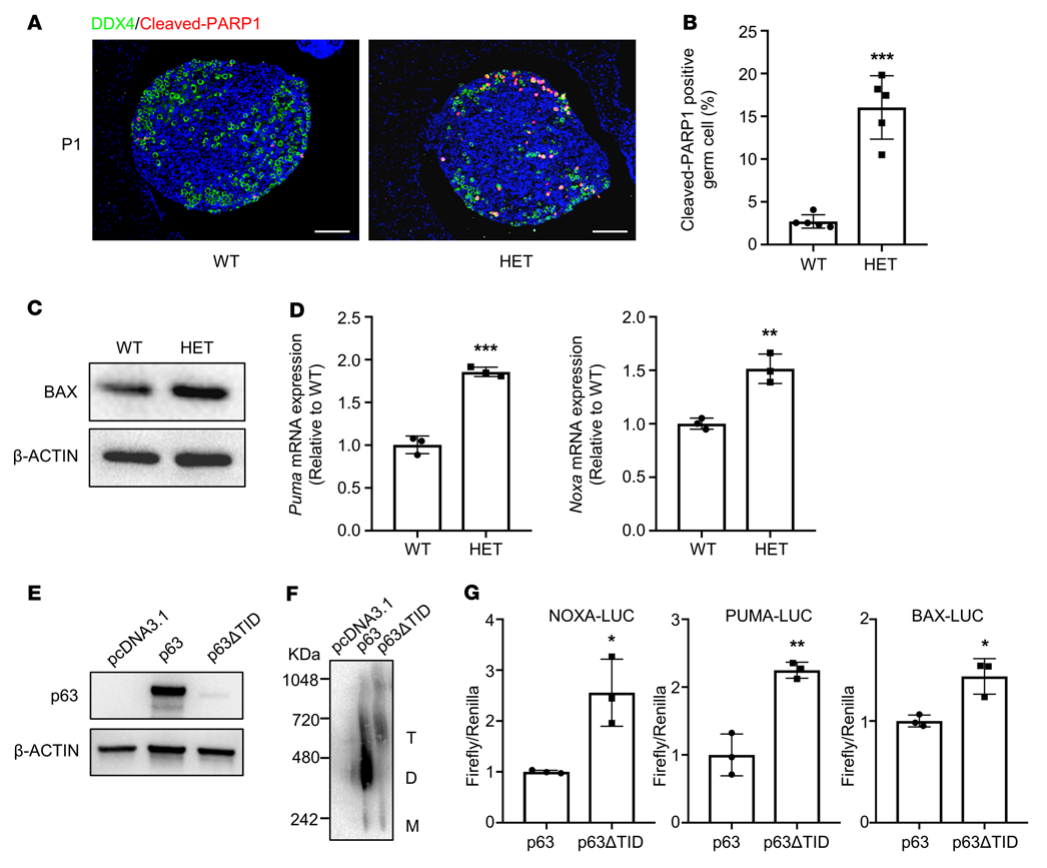
The oocytes in *p63^+/ΔTID^* mouse ovaries died by apoptosis. (**A**) IF staining for DDX4 (green) and cleaved-PARP1 (red) in ovary sections from P1 WT and *p63*^+/ΔTID^ mice. Cell nuclei were counterstained with DAPI (blue). Scale bars: 50 μM. (**B**) Quantitative analysis of cleaved-PARP1–positive oocytes in each group (*n* = 5). (**C**) Western blot analysis of BAX expression in P1 WT and *p63*^+/ΔTID^ ovaries. β-Actin was used as the loading control. (**D**) Quantitative RT-PCR analysis of *Puma* and *Noxa* gene expression in P1 ovaries of WT and *p63*^+/ΔTID^ mice. *Gapdh* was used as the internal control. Three mice for each genotype were used for each independent experiment, and 3 independent experiments were conducted. (**E**) The *p63* and *p63*Δ*TID* plasmids were transfected into SAOS-2 cells, and protein levels were detected by Western blotting. β-Actin was used as the loading control. (**F**) Oligomeric state analysis of p63 and p63ΔTID using BN-PAGE. The oligomeric state is indicated by T, D, and M. (**G**) Transcriptional activity of p63ΔTID in SAOS-2 cells on the *NOXA*, *PUMA*, and *BAX* promoters. The activity of the p63 group was set to 1, and 3 independent experiments were conducted. In **B**, **D** and **G**, data are shown as the mean ± SD. *P < 0.05, **P < 0.01, and ***P < 0.001, by unpaired, 2-tailed Student’s *t* test.

To further investigate the underlying molecular mechanism of rapid oocyte loss, we examined cell apoptosis in mouse ovaries. IF staining of P1 ovarian sections showed that cleaved-PARP1–positive oocytes were significantly increased in *p63*^+/ΔTID^ ovaries (Figure 5, A and B). As expected, increased expression of BAX protein was detected in P1 *p63*^+/ΔTID^ ovaries (Figure 5C). The mRNA levels of 2 target genes of p63, *Puma* and *Noxa,* were also significantly increased (Figure 5D). These results suggested that deleting the TID of the p63 protein was sufficient to induce uncontrolled apoptosis of the oocytes in primordial follicles without exogenous damage.

To verify whether p63ΔTID was autoactivated in vitro, we induced overexpression of mouse WT p63 and mutant p63ΔTID proteins in SAOS-2 cells. The results revealed that the p63ΔTID protein was almost undetectable by Western blotting (Figure 5E) and formed an activated tetramer after inhibition of proteasome-dependent degradation, as shown by the BN-PAGE analysis (Figure 5F). The p63ΔTID protein also transcriptionally activated the *PUMA*, *NOXA*, and *BAX* promoters compared with WT p63, as detected by the luciferase reporter assays (Figure 5G). In summary, these results confirmed that activated p63ΔTID triggered downstream proapoptotic pathways and thus caused oocyte exhaustion and infertility.

### p63^+/R647C^ mice also display a POI-like phenotype.

**Figure 6:**
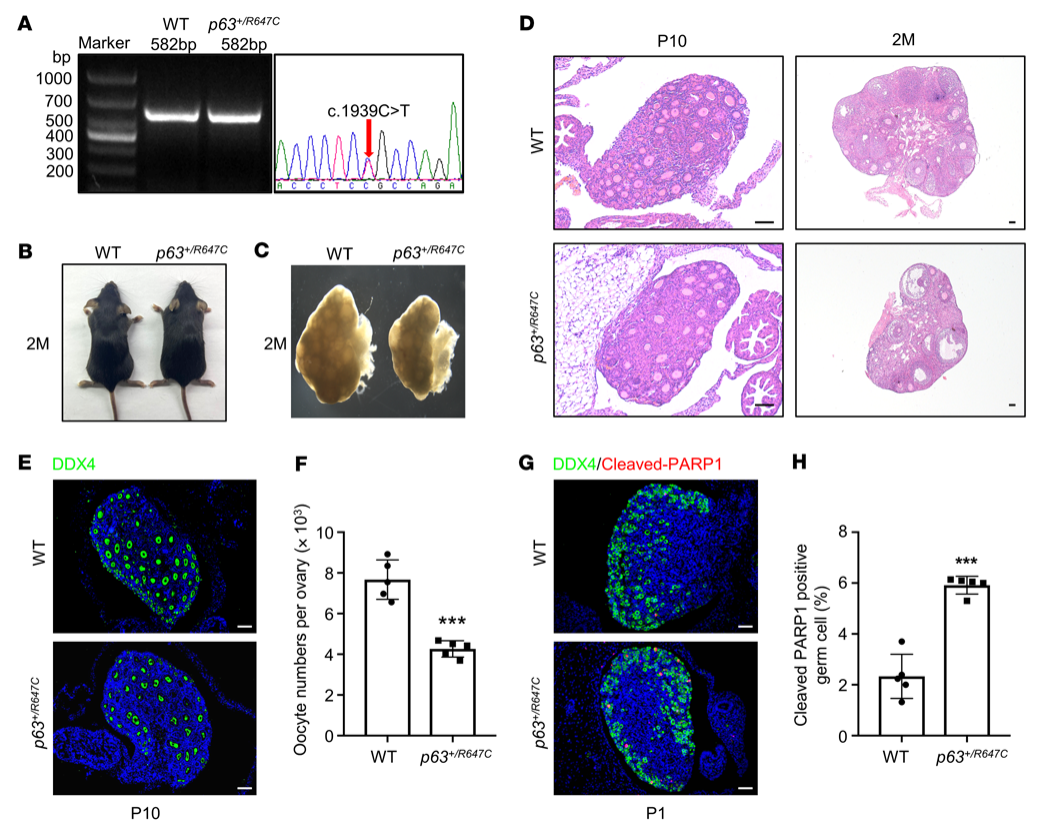
Accelerated oocyte loss in *p63^+/R647C^* mice by apoptosis. (**A**) Validation of the genotype of the *p63^+/R647C^* mouse by Sanger sequencing. (**B**) Gross morphology of 2M WT and *p63^+/R647C^* females. (**C**) Sizes of ovaries from 2M WT and *p63^+/R647C^* females. (**D**) H&E staining of ovary sections from P10 and 2M WT and *p63^+/R647C^* mice. Scale bars: 50 μM. (**E**) IF staining for DDX4 (green) in P10 WT and *p63^+/R647C^* ovaries. Cell nuclei were counterstained with DAPI (blue). Scale bars: 50 μM. (**F**) Quantitative analysis of DDX4-expressing oocytes from mice in each group. Data are presented as the mean ± SD. *n* = 5. ***P < 0.001, by unpaired, 2-tailed Student’s *t* test. (**G**) IF staining for DDX4 (green) and cleaved-PARP1 (red) in ovary sections from P1 WT and *p63^+/R647C^* mice. Cell nuclei were counterstained with DAPI (blue). Scale bars: 50 μM. (**H**) Quantitative analysis of cleaved-PARP1–positive oocytes from mice in each group. Data are shown as the mean ± SD. *n* = 5. ***P < 0.001, by unpaired, 2-tailed Student’s *t* test.

To further validate the functional effect of TID-related missense mutations in vivo, we generated a *p63-*mutant mouse model carrying p.R647C, which was found in 3 unrelated patients (Table 1 and Figure 6A). The *p63^+/R647C^* mice were viable at birth, and no obvious developmental defects were observed at 2 months of age (Figure 6B). However, their ovary size was obviously reduced compared with that of WT females (Figure 6C), and the number of follicles was substantially reduced in P10 and 2M *p63^+/R647C^* ovaries (Figure 6D). The number of oocytes with DDX4 staining in P10 *p63^+/R647C^* mice was reduced to approximately 50% of that in WT mice (Figure 6, E and F). Furthermore, cleaved-PARP1–positive oocytes were significantly increased in P1 *p63^+/R647C^* ovaries compared with WT ovaries (Figure 6, G and H). These results showed that the TID point mutation also led to accelerated oocyte loss via apoptosis after birth. However, in comparison with *p63*^+/ΔTID^ mice, the rate of follicular depletion was slower, and more follicles were retained in adulthood. This was consistent with the phenotype of the secondary amenorrhea seen in the 3 patients with POI carrying the p.R647C mutation.

**Figure 7:**
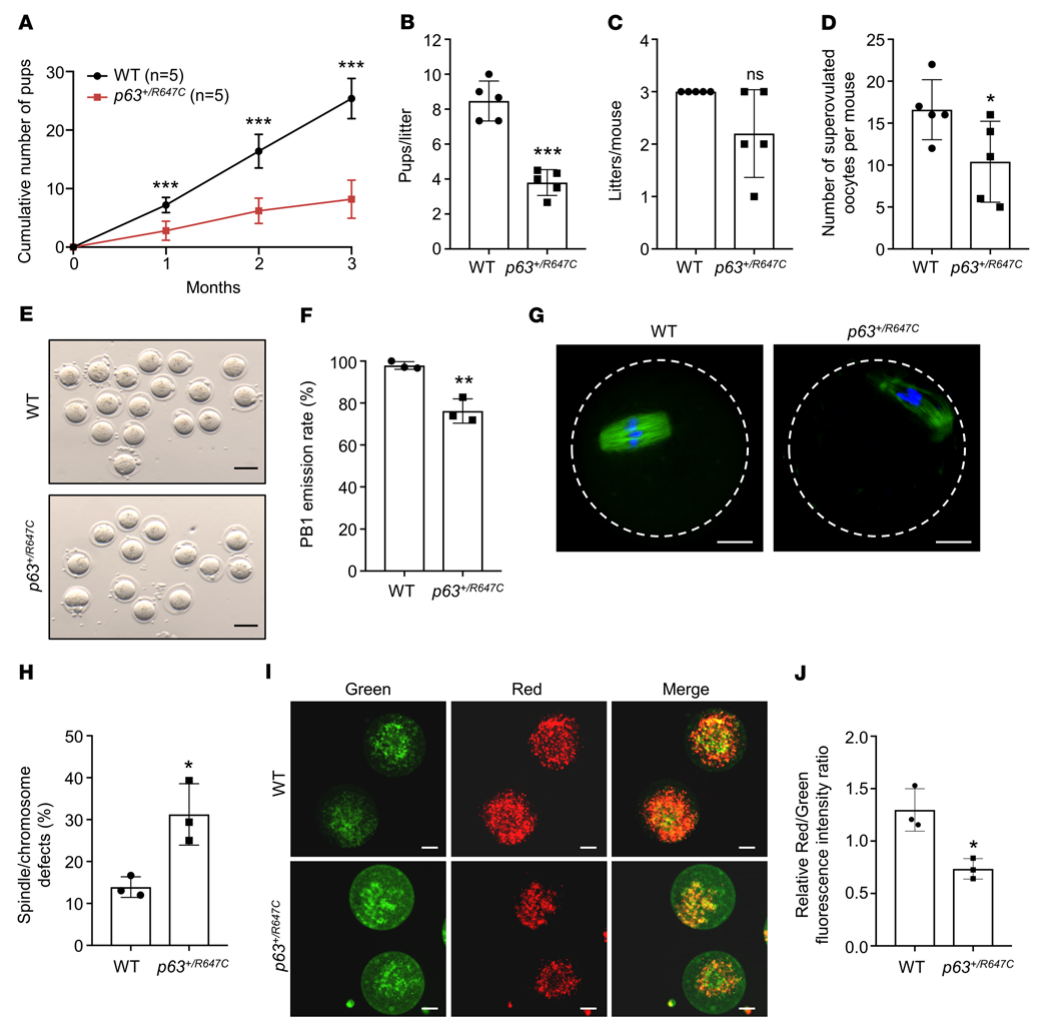
Evaluation of fertility and oocyte quality in *p63^+/R647C^* mice. (**A**) Cumulative number of pups obtained from WT females (*n* = 5) and *p63^+/R647C^* females (*n* = 5) crossed with WT males for a period of 3 months. (**B** and **C**) The numbers of pups per litter and the number of litters per mouse were recorded for each group (*n* = 5) in the fertility test. (**D**) Number of superovulated oocytes per mouse obtained from the 2 groups (*n* = 5 for each genotype). (**E**) Morphology of superovulated oocytes obtained from 3-week-old WT and *p63^+/R647C^* mice. Scale bars: 100 μm. (**F**) Percentages of MII oocytes with PB1 emission in WT and *p63^+/R647C^* oocytes. Three mice for each genotype were used for each independent experiment, and 3 independent experiments were conducted. (**G**) The morphology of spindle and chromosome organization in WT and *p63^+/R647C^* oocytes. Anti–α-tubulin antibody (green) was used to stain the spindles. Chromosomes were counterstained with DAPI (blue). Scale bars: 20 μm. (**H**) Percentages of oocytes with spindle/chromosome defects in WT and *p63^+/R647C^* oocytes. Three mice of each genotype were used for each independent experiment, and 3 independent experiments were performed. (**I**) Oocyte MMP shown by JC-1 staining in the 2 groups. Red fluorescence indicates JC-1 aggregates with higher MMP, and green fluorescence indicates JC-1 monomers with lower MMP. Scale bars: 20 μm. (**J**) Quantification of the ratio of red to green fluorescence intensity in the 2 groups. Three mice for each genotype were used for each independent experiment, and 3 independent experiments were performed. (**A**–**D**, **F**, **H**, and **J**) Data are presented as the mean ± SD. *P < 0.05, **P < 0.01, ***P < 0.001, by unpaired, 2-tailed Student’s *t* test.

Finally, we analyzed the fertility and quality of the remaining oocytes in *p63^+/R647C^* mice. Although the *p63^+/R647C^* mice were fertile, the cumulative numbers of pups and pups per litter were significantly reduced in *p63^+/R647C^* mice compared with those of WT controls (Figure 7, A–C). After superovulation of 3-week-old mice, the number of harvested oocytes per mouse and the first polar body (PB1) emission rates were significantly decreased in the mutant mice (Figure 7, D–F). Furthermore, the percentage of superovulated oocytes with abnormal spindle/chromosomes was significantly increased in the mutant mice (Figure 7, G and H), and JC-1 staining showed lower mitochondrial membrane potential (MMP) in the mutant oocytes than in WT controls (Figure 7, I and J). Taken together, these results demonstrated that the quality of the remaining oocytes in the *p63^+/R647C^* mice was impaired. Both the reduced numbers and poor quality of the oocytes probably contributed to the decreased fertility of the *p63^+/R647C^* mice.

## Discussion

The TAp63α protein is a quality control factor for determining the fate of oocytes with damaged genomes (11, 24). Recently, *TP63* was highlighted as a gene associated with age at natural menopause in a large population study (36), suggesting that it might regulate the depletion rate of primordial follicles and thus shape the ovarian reserve. Furthermore, *TP63* mutations affecting the C-terminal TID have been reported in different populations of patients with syndromic or isolated POI (26–29), but they were identified only in a limited number of patients, and the contribution of *TP63* to POI pathogenesis needs to be further determined. In this study, we found that 6 TID-related C-terminal mutations of *TP63* were causative for POI and could explain 0.78% (8 of 1,030) of the cases of isolated POI in a large Chinese population sample. This finding indicates that *TP63* is a relatively common POI-causative gene and should be included in genetic screening for patients with POI.

Because of the complexity of the genotype-phenotype correlation of *TP63* gene mutations, the specific effects of TID-related variants identified in patients with POI need to be further determined. In this study, we show that the heterozygous mutations impairing the TID of TAp63α were sufficient to cause isolated POI, which is supported by both in vitro functional studies and *p63*-mutant mouse models. Deletion of the entire TID caused intense activation of TAp63α, and *p63*^+/ΔTID^ mice exhibited a complete loss of oocytes shortly after birth, which recapitulated the phenotype of primary amenorrhea in patients with POI carrying TID truncated mutations (Supplemental Table 1) (28). It is worth noting that 4 missense mutations (p.R643Q, p.L646P, p.R647C, and p.R655Q) located in the core sequence of the TID were also identified in patients with POI. These mutations attenuated the interaction between the TID and the TAD (Figure 1, D and E), resulting in an activated tetrameric conformation of TAp63α that exerted proapoptotic effects. Furthermore, *p63^+/R647C^*-mutant mice showed accelerated oocyte loss, reduced fertility, and impaired oocyte quality, but the phenotypes were less severe than those of *p63*^+/ΔTID^ mice. Consistent with this, the 3 patients with POI carrying the p.R647C point mutation all presented with secondary amenorrhea (Supplemental Table 1).

In addition to the direct interaction between the TID and the TAD, posttranslational sumoylation at the p63 C-terminal lysine residue K637 (K676 in NM_003722.5) also had an inhibitory effect by reducing the p63 protein concentration (16, 37). While the truncated mutations removed the 2 inhibitory mechanisms, the point mutations only affected the former. Therefore, different TID-related variations with distinct transactivation capacities might determine the rate of oocyte loss and consequent phenotypic variability. In addition, all patients in this study manifested isolated POI, which was consistent with the phenotypes of *p63*^+/ΔTID^ and *p63^+/R647C^* mice, verifying that *TP63* mutations can cause an isolated germline phenotype. All of the POI-related *TP63* mutations identified to date are heterozygous mutations, and homozygous perturbations of *TP63* remain to be identified in patients with POI and might cause a more severe phenotype.

Under physiological conditions, TAp63α is maintained in an inactive dimer in which the TID and TAD interact by forming a 6-stranded antiparallel β-sheet (17, 18). In this study, we first demonstrated that loss of just the p63 TID was sufficient to deplete oocytes in vivo, and then we compared this with the C-terminal sequences of different knockout strategies (Supplemental Table 2). While Suzuki et al. deleted the entire SAM and TID domains generating Cα′ (38), Lena et al. deleted exon 13 of the *p63* gene, resulting in the replacement of p63α with p63β, which lacks most of the SAM domain and the entire TID (30). In contrast, we specifically deleted the TID and preserved the integrity of the SAM domain (CαΔTID). The Cα′ terminus was the shortest construct, followed by Cβ and then CαΔTID. While the Cβ and CαΔTID mutants were autoactivated in the oocytes and caused infertility in mice (30), the heterozygous mice with Cα′ were fertile (38), indicating that the p63 protein with Cα′ was not spontaneously activated in oocytes. Taken together, we speculated on the existence of a domain that is essential for activation in oocytes (EAO) in the TAp63α protein (Supplemental Table 2). Consistent with this hypothesis, all of the POI-related mutations identified in both our current study and in previous studies are downstream of the EAO domain (Figure 1A). Recently, 2 truncated mutations of TAp63α — DelACTT1217 (c.1334_1337delACTT, p.L446Ffs*20 in NM_003722.5) and ΔExon11 — were predicted to cause oocyte apoptosis (30). However, the 2 mutations are located upstream of the EAO domain and cause loss of the domain, which might not affect female fertility. Therefore, we further refined the prediction strategy for oocyte fate and emphasized an autoinhibitory state of TAp63α in the oocyte. It can be concluded that both the truncated variations downstream of the EAO domain and the point mutations localized in the core sequence of the TID generate constitutively active mutants and cause premature depletion of oocytes. In addition, a missense mutation, p.R97P, located in the N-terminal TAD was also identified in a patient with POI, which also led to tetramer formation and constitutive activation of the mutant TAp63α protein (31), suggesting that N-terminal mutations, which only disrupt the interaction of the TAD and TID, but do not impair the transactivation activity of TAD, can also cause POI.

Although we previously revealed the role of *TP63* mutations in the 3′–UTR in human Müllerian duct anomalies (39), neither the patients with POI nor the mouse models in this study showed defects in the reproductive tract, which might be due to the differences in mutation sites/types and the expression of tissue-specific isoforms. There is also evidence that p63 functions in spermatogenesis in males. TAp63 was reported to be involved in recombination-dependent pachytene arrest in mouse spermatocytes (40), and increased apoptosis of male germ cells was observed in *p63^+/−^* mice (41). However, both *p63*^+/ΔTID^ and *p63^+/R647C^* male mice, as well as the Δ13p63 heterozygous mice reported by Lena et al. (30), were fertile, indicating that there was no obvious abnormality in spermatogenesis in these mice. Consistently, the father of the patient with POI harboring the p.R643Q mutation in *TP63* was fertile (Supplemental Figure 1B). These results indicate that the TID-related mutations in *TP63* might only affect female fertility, and tissue-specific functions of p63 might reflect different strategies for controlling the quality of female and male germ cells.

ΔNp63α, which also has a TID, is highly expressed in epithelial tissues and regulates epithelial morphogenesis and development (9). It has been proposed that the C-terminus of ΔNp63α contains multiple acceptor sites for posttranslational modifications, such as sumoylation and ubiquitylation, which together ensure the efficient degradation of ΔNp63α (42, 43). The C-terminal mutation p.Q634* (p.Q673* in NM_003722.5) in *TP63* was found in patients with split-hand/split-foot malformations, with increased transcriptional activity on the promoters of several skin-specific genes (44). In addition, patients with syndromic POI carrying the p.R643* mutation also exhibited nipple anomalies and lacrimal duct atresia (29). These cases imply that the C-terminal TID can also regulate the function of the ΔNp63α protein. However, both *p63*^+/ΔTID^ and *p63^+/R647C^* mice in our study, as well as the reported Δ13p63 heterozygous mice (30), had no apparent morphological defects other than POI. Similarly, no somatic phenotypes were observed in the patients with POI whom we studied. These findings indicate that the loss or mutation of the TID does not obviously affect the function of ΔNp63α or the development of epithelial tissues. Therefore, the correlations between somatic phenotypes and *TP63* C-terminal mutations need further exploration.

In conclusion, we identified what we believe to be novel gain-of-function mutations in *TP63*, determined the contribution of these mutations to POI, and further demonstrated the essential role of the p63 C-terminus in maintaining the ovarian reserve. Our results verify that *TP63* is a causative gene for isolated POI, and this finding, we believe, has significant clinical implications. While our findings are instructive for genetic counseling and child-bearing plans in female carriers with *TP63* mutations, male carriers who are fertile should also seek genetic counseling and appropriate interventions such as preimplantation genetic testing before having children. Furthermore, the development of strategies that inhibit TAp63α activation in oocytes might be helpful for prolonging the female reproductive lifespan.

## Methods

### Study participants.

A total of 1,030 Chinese patients with idiopathic POI were enrolled in this WES project. The diagnostic criteria were primary or secondary amenorrhea before 40 years of age, along with serum FSH levels above 25 IU/L on 2 occasions more than 4 weeks apart. Patients with chromosomal abnormalities, a history of ovarian surgery, previous chemo- or radiotherapy, or autoimmune disorders were excluded (45).

### WES and variant analysis.

Genomic DNA was extracted from peripheral blood following standard procedures using the DNeasy Blood and Tissue Kit (QIAGEN). Genomic DNA from all patients with POI in this study was evaluated by WES on the Illumina HiSeq platform, and the data were analyzed as described previously (45). The sequencing data for the 1,030 patients with POI in this study are publicly accessible in the Genome Sequence Archive at the National Genomics Data Center (NGDC) (https://ngdc.cncb.ac.cn/gsa-human/) under accession number HRA003245. The variants present in *TP63* predicted to be damaging were corroborated by Sanger sequencing. Their pathogenicity was evaluated according to the American College of Medical Genetics and Genomics guidelines (34). The specific primers used for PCR amplification are shown in Supplemental Table 3.

### Cell culturing, plasmid construction, and transfection.

The human osteosarcoma cell line SAOS-2 (National Collection of Authenticated Cell Cultures) and HEK293 cells (ATCC) were grown in DMEM (Gibco, Thermo Fisher Scientific) containing 10% FBS (Gibco, Thermo Fisher Scientific) and 1% penicillin-streptomycin (Invitrogen, Thermo Fisher Scientific) at 37°C with 5% CO_2_. The expression plasmid for human *TP63* used in this study has been described previously (39). POI-related *TP63* mutants were constructed from the WT pcDNA3.1-*TP63* plasmid using the QuikChange Lightning Site-Directed Mutagenesis Kit (Agilent Technologies). Transient transfection was performed with Lipofectamine 3000 Transfection Reagent (Invitrogen, Thermo Fisher Scientific) according to the manufacturer’s protocol.

### Luciferase reporter assay.

The promoter fragments of *PUMA*, *NOXA*, and *BAX* were amplified from human genomic DNA and cloned into the pGL3-basic vector to generate firefly luciferase reporter constructs. The SAOS-2 cells were plated into 12-well plates and cotransfected with 0.65 μg pGL3-*PUMA*, pGL3-*NOXA*, or pGL3-*BAX* reporter constructs, 0.1 μg pRL-TK *Renilla* luciferase plasmid, and 0.25 μg WT or mutant *TP63* plasmids. After 36 hours, cells were processed, and luciferase activity was detected by a multimode plate reader (PerkinElmer) using the Dual-Luciferase Reporter Assay System (Promega, catalog E1910) according to the manufacturer’s instructions. The results were obtained by calculating the ratio of firefly luciferase activity to *Renilla* luciferase activity, and the experiments were performed independently in triplicate.

### Western blotting.

For the in vitro experiments, cells were lysed in SDS buffer (Beyotime) with protease inhibitor cocktail (Cell Signaling Technology) 48 hours after transfection. For mouse samples, total protein from WT and mutant mouse ovaries was extracted with a Minute Total Protein Extraction Kit (Invent Biotechnologies) according to the manufacturer’s instructions. Equal amounts of protein samples were subjected to 10% SDS-PAGE and transferred onto a PVDF membrane (MilliporeSigma). The membrane was blocked with 5% nonfat milk in TBST for 1 hour at room temperature. The following primary antibodies were used for immunoblotting: anti-p63 (1:1,000 dilution, Abcam, catalog ab124762); anti-BAX (1:1,000 dilution, Cell Signaling Technology, catalog 2772S); anti–β-actin (1:1,000 dilution, proteintech, catalog 66009-1-lg); and anti-GFP (1:1,000 dilution, Abcam, catalog ab290). After incubation at 4°C overnight with gentle agitation, the secondary goat anti–rabbit or anti–mouse IgG-HRP antibodies were applied (1:5,000 dilution, Proteintech, catalogs SA00001-2 and SA00001-1). Proteins were detected with an ECL kit (MilliporeSigma) and visualized using the ChemiDoc MP System (Bio-Rad). Quantification of band signals was analyzed by ImageJ software (NIH).

### BN-PAGE.

BN-PAGE analysis was performed as described previously (30). After treatment with 10 μM MG132 (Merck, catalog 474790) for 12 hours, the transfected SAOS-2 cells were lysed and incubated for 30 minutes on ice with the NativePAGE Sample Prep Kit (Thermo Fisher Scientific, catalog BN2008) supplemented with protease and phosphatase inhibitors (Thermo Fisher Scientific, catalog 78442) and benzonase (Merck Millipore). Protein samples were centrifuged at 20,000*g* for 30 minutes at 4°C. The supernatant was collected, and Coomassie G-250 was added at 1/4 detergent (10% DDM) concentration. The samples were separated on a NativePAGE Novex 3%–12% Bis-Tris protein gel system (Thermo Fisher Scientific, catalog BN1003BOX) according to the manufacturer’s instructions. In brief, the nondenaturing electrophoresis was performed with 1× NativePAGE Running Buffer (Thermo Fisher Scientific, BN2001) and blue cathode buffer (containing 0.002% G-250). Proteins were transferred onto PVDF membranes in 1× NuPAGE Transfer Buffer (Thermo Fisher Scientific, catalog NP00061). The membrane was incubated for 15 minutes in 8% acetic acid and destained using 100% methanol. The membrane was then blocked with 5% nonfat milk in TBST and incubated overnight with anti-p63 primary antibody (1:1,000 dilution, Abcam, catalog ab124762) followed by a 1-hour incubation with the secondary goat anti–rabbit IgG-HRP antibody (1:5,000 dilution, Proteintech, catalog SA00001-2) and detection with an ECL kit (MilliporeSigma).

### TUNEL assay.

SAOS-2 cells were transfected with WT or mutant *TP63* expression plasmids. After 48 hours, the cells were fixed with 4% paraformaldehyde (PFA), and a TUNEL assay was performed using the In Site Apoptosis Detection Kit (KeyGEN, catalog KGA7063) according to the manufacturer’s recommendations. Images were captured under a fluorescence microscope (Olympus). The percentages of TUNEL-positive cells were recorded in at least 5 fields, and 3 independent experiments were conducted.

### Co-IP experiments.

Plasmids containing TAp63αΔTID and WT or mutant GFP-TID were cotransfected into HEK293 cells for 36 hours and then treated with 10 μM MG132 (Merck, catalog 474790) for 12 hours. The cells were washed twice with cold PBS and lysed in IP lysis buffer (Thermo Fisher Scientific, catalog 87787) with 1% protease inhibitor cocktails (Thermo Fisher Scientific, catalog 78442) on ice for 30 minutes. The lysates were sonicated to release nuclear proteins and then centrifuged at 14,000*g* for 10 minutes to obtain the supernatant. The protein concentration was determined with a BCA kit (Thermo Fisher Scientific, catalog 23225). The samples as input were directly separated for detection. A total of 0.5 mg protein extracts was incubated with anti-p63 antibody (Cell Signaling Technology, catalog 39692) or rabbit IgG antibody (Cell Signaling Technology, catalog 3900) and Protein A/G Magnetic Beads (MilliporeSigma, catalog LSKMAGAG02) at 4°C overnight with slow rotation. The beads were washed 3 times with IP lysis buffer. The immunoprecipitates were then added to the loading buffer, denatured for 6 minutes at 100°C, and subjected to Western blotting. The experiments were conducted 3 times.

### Generation of p63^+/ΔTID^ and p63^+/R647C^ mice.

The *p63*^+/ΔTID^ mice were generated using standard embryonic stem cell (ESC) technology (Cyagen Biosciences). The targeting vector was designed to contain 2 homologous arms to facilitate effective homologous recombination, which was generated by PCR using BAC clones as templates. The c.1828_1829insGA (p.F610*, NM_001127259.1) point mutation was introduced into exon 14, resulting in the formation of a premature stop codon and thus the loss of the TID domain. Targeted ESC clones were injected into C57BL/6 albino embryos to generate chimeras. Their germline transmission was confirmed by breeding with C57BL/6 females and subsequent genotyping of the offspring. Mice carrying the *p63* c.1939C>T (p.R647C, NM_001127259.1) point mutation were generated using the CRISPR/Cas9 system (Shanghai Model Organisms Center, Shanghai, China), and heterozygous *p63^+/R647C^* mice were obtained by intercrossing of male *p63^+/R647C^* and female WT mice. Genotyping was assessed by PCR and Sanger sequencing using mouse tail genomic DNA (primers are listed in Supplemental Table 3). All mice were housed under specific pathogen–free conditions with a 12-hour light/12-hour dark cycle and free access to food and water. Mice of the appropriate genotype were mated at night, and noon on the day after a vaginal plug was seen was considered E0.5.

### Fertility test.

For fertility testing of *p63*^+/ΔTID^ mice, each adult heterozygous male or female mouse was mated with age-matched WT C57BL/6J females or males for 6 months. For *p63^+/R647C^* mice, WT and *p63^+/R647C^* adult females were mated with age-matched C57BL/6J males for 3 months. The number of live-born pups in each cage was counted individually.

### Histology and IF.

For histological analysis, mouse ovaries were fixed in Bouin’s solution overnight, dehydrated in an ethanol gradient, embedded in paraffin, and cut into 5 μm sections. The sections were then deparaffinized, rehydrated, and stained with H&E following standard procedures. For IF experiments, WT and *p63*^+Δ/TID^ (or *p63^+/R647C^*) ovaries or skin samples were fixed in 4% PFA, embedded in paraffin, and sectioned at 5 μm thickness. The slides underwent antigen retrieval and were then blocked, followed by incubation at 4°C overnight with the following primary antibodies: anti-DDX4 (1:600 dilution, Abcam, catalog ab27591); anti-p63 (1:200 dilution, Abcam, catalog ab124762); anti–cleaved-PARP1 (1:400 dilution, Cell Signaling Technology, catalog 94885S); anti-K10 (1:150 dilution, Abcam, catalog ab76318); anti-K14 (1:100 dilution, Santa Cruz Biotechnology, catalog sc-53253); and anti-Ki67 (1:200 dilution, Abcam, catalog ab15580). The slides were then washed in PBS, incubated with secondary antibodies (Alexa Fluor 568–conjugated donkey anti-rabbit antibody or Alexa Fluor 488–conjugated donkey anti-mouse antibody, 1:800 dilution, Invitrogen, Thermo Fisher Scientific, catalogs A21202 and A10042) for 1 hour at room temperature, and then sealed in antifade fluorescence mounting medium (Abcam). Images were captured with a fluorescence microscope (Olympus) and analyzed with ImageJ.

### Oocyte quantification.

The 5 μm serial mouse ovary sections were stained with anti-DDX4 antibody, and oocytes were counted in every fifth section. The sum of oocytes counted was multiplied by 5 to estimate the total number of oocytes per ovary.

### RNA extraction and RT-PCR.

For the quantitative RT-PCR experiment, a total of 6 ovaries were rapidly isolated from 3 P1 mice of identical genotypes and pooled together for RNA extraction. Total RNA was isolated using an RNeasy Mini Kit (QIAGEN) and reverse-transcribed into cDNA using the PrimeScript RT reagent kit (Takara, catalog RR047A). Real-time PCR was performed with SYBR Green I Master Mix (Roche, catalog 4707516001) on a LightCycler 480 system (Roche). The relative expression of the target genes was normalized to *Gapdh* and calculated using the 2^−ΔΔCt^ method. The primers used for real-time PCR are listed in Supplemental Table 3. Three independent experiments were conducted. For the RT-PCR experiment, total RNA was extracted from the skin, ovary, hypothalamus, and pituitary of each mouse using an RNeasy Mini Kit (QIAGEN) and was reverse transcribed into cDNA with a PrimeScript RT Kit (Takara, catalog RR047A). Semiquantitative PCR was performed with 2× Accurate Taq Master Mix (Accurate Biology, catalog AG11010), and this was followed by agarose gel electrophoresis. The PCR primers designed by Lena et al. are listed in Supplemental Table 3 (30). For quantitative PCR of hypothalamus and pituitary tissues, the reagents and methods used were the same as those described above. The primers are listed in Supplemental Table 3 with reference to the previous study (46).

### Oocyte collection and IF.

Female mice (*n* = 3 for each genotype) at 3 weeks of age were intraperitoneally injected with 5 IU pregnant mare’s serum gonadotropin (PMSG) (Ningbo Sansheng Pharmaceutical) followed by human chorionic hormone (hCG) (Ningbo Sansheng Pharmaceutical) 44 hours later. Oocytes were collected from the oviducts 14 hours after hCG injection, and the PB1 emission was observed with a stereoscope (Nikon, SMZ1500). For IF staining, oocytes were fixed in 4% PFA for 30 minutes at room temperature following permeabilization in PBS containing 0.5% Triton X-100 for 30 minutes. Oocytes were then blocked in PBS containing 1% BSA for 1 hour and incubated with anti–α-tubulin–FITC antibody (1:500 dilution, MilliporeSigma, catalog F2168) for 1.5 hours at room temperature. After washing in PBS, oocytes were shifted onto glass slides, and the slides were sealed with Mounting Medium with DAPI (Abcam, catalog ab104139). Images were acquired with a Dragonfly spinning disc confocal microscope (ANDOR Technology). The experiments were performed independently in triplicate.

### Oocyte MMP measurement.

The measurement of oocyte MMP was performed according to the instructions of the JC-1 assay kit (Yeasen, catalog 50101ES01). Briefly, the collected MII oocytes from superovulated mice (*n* = 3 for each genotype) were washed 3 times with M2 medium (MilliporeSigma, catalog M7167) and then incubated for 30 minutes with staining solution in M16 medium (MilliporeSigma, catalog M7292) in the dark in a cell culture incubator. The oocytes were then washed 3 times in JC-1 buffer solution. Images were acquired with a Dragonfly spinning disc confocal microscope, and the relative fluorescence intensity was quantified with ImageJ. Three independent experiments were conducted.

### Intraperitoneal glucose tolerance test.

For the intraperitoneal glucose tolerance test (IGTT), mice were intraperitoneally injected with glucose at 2 g/kg body weight after a 16-hour fast. Blood glucose was analyzed using tail blood before and 15, 30, and 60 minutes after glucose injection. AUCs were calculated using GraphPad Prism 8.0 (GraphPad Software).

### Serum triglyceride and total cholesterol measurements.

Blood collected from mice that had been fasted for 16 hours was kept at room temperature for 2 hours and then centrifuged at 1,000*g* for 5 minutes to obtain the upper serum layer. Serum triglyceride and total cholesterol measurements were conducted using the Liquid Sample Triglyceride Content Assay Kit (Applygen, catalog E1003) and the Liquid Sample Total Cholesterol Content Assay Kit (Applygen, catalog E1005), respectively.

### Statistics.

Data were analyzed using SPSS 21.0 and GraphPad Prism 8.0. Data are presented as the mean ± SD. The numerical results between 2 groups were compared by 2-tailed Student’s *t* test or 1-way ANOVA followed by Dunnett’s test. A *P* value of less than 0.05 was considered significant.

### Study approval.

Written informed consent was obtained from each participant, and the study was approved by the IRB of the Center for Reproductive Medicine of Shandong University. All animal studies were performed in accordance with guidelines approved by the IACUC of Shandong University.

## Author contributions

CZH performed the experiments, analyzed the results, and wrote the manuscript. SMZ and XM conducted the experiments. HNK and TG analyzed the sequencing data. YJY provided guidance and contributed to the writing of the manuscript. SDZ, YYQ, and ZJC designed and supervised the study and wrote the manuscript. The order of the names of the co–first authors was determined on the basis of CZH’s contributions the work (leading the majority of the experiments performed and writing of the manuscript).

## Supplementary Material

Supplemental data

## Figures and Tables

**Table 1:**
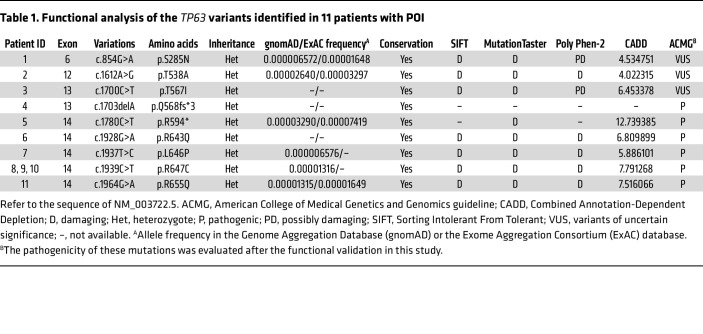
Functional analysis of the *TP63* variants identified in 11 patients with POI

## Version 1 03/01/2023

Electronic publication
