# Supplementary material for: TP63 gain-of-function mutations cause premature ovarian insufficiency by inducing oocyte apoptosis
Source: J Clin Invest. 2023 Mar 1;133(5):e162315. doi: 10.1172/JCI162315 (PMC9974095; doi:10.1172/JCI162315)
Supplement: Supplemental data [file jci-133-162315-s022.pdf]

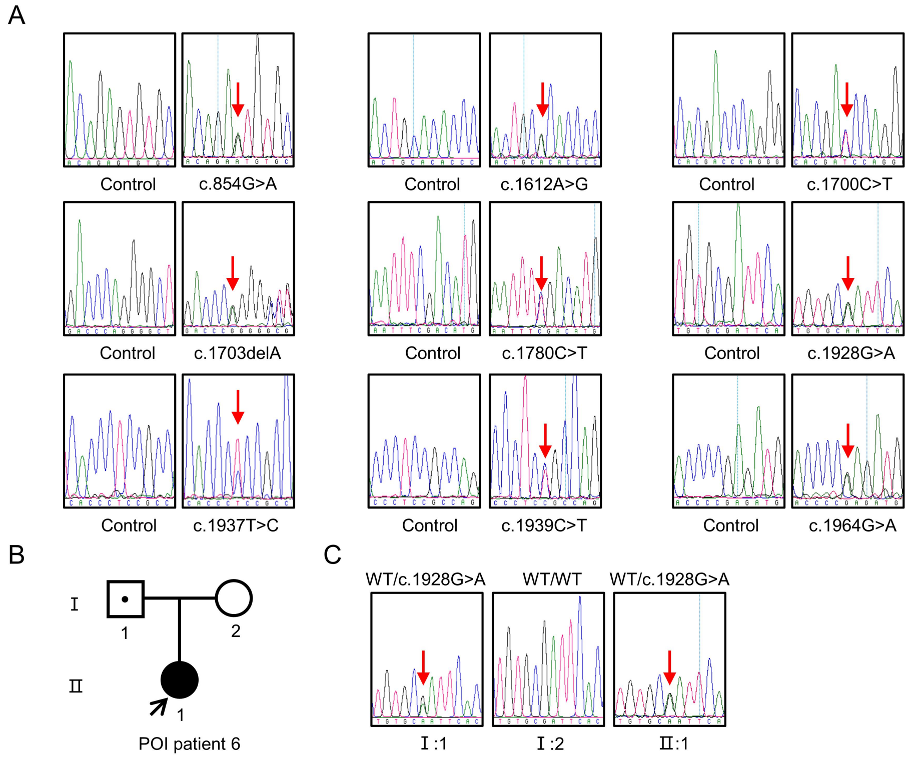

Supplemental Figure 1. Validation of the *TP63* mutations in POI patients. (A) Sequence chromatograms showing nine heterozygous variants in POI patients that were not present in the controls. NM_003722.5 was used as the reference sequence. **(B and C)** Sanger sequencing confirmed the presence of the c.1928G>A variant in POI patient 6 and her father, but not in her mother.

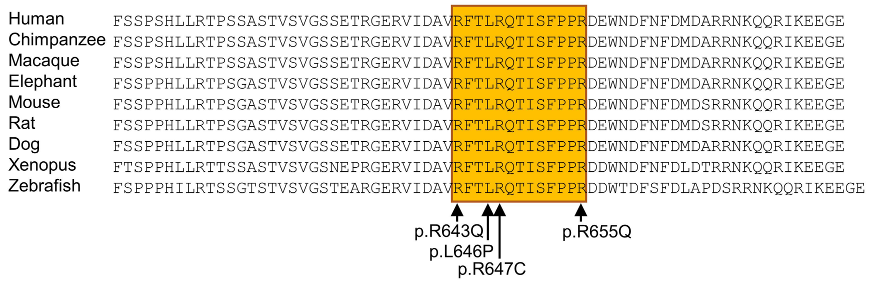

Supplemental Figure 2. Conservation analysis of amino acids in the TID among various species. The yellow frame indicates the core sequence of the TID. The positions of the four novel point mutations in the core sequence are shown.

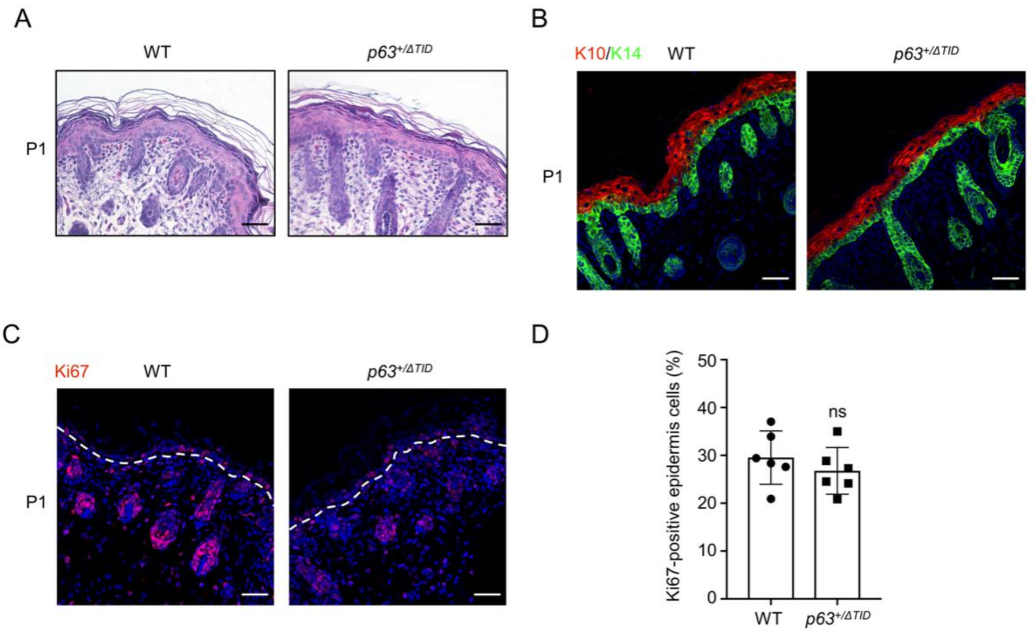

Supplemental Figure 3. Epidermis characterization of *p63*$^{+/\Delta TID}$ mice. (A) HE staining of dorsal skin sections of P1 WT and *p63*$^{+/\Delta TID}$ mice. Scale bar: 50 μM. **(B and C)** IF staining of K10 (red), K14 (green), and Ki67 (red) on dorsal skin sections of P1 WT and *p63*$^{+/\Delta TID}$ mice. Cell nuclei were counterstained with DAPI (blue). Scale bar: 50 μM. **(D)** Quantitative analysis of Ki67-positive cells in the epidermis. Data are shown as the mean ± SD, n = 6 for each genotype. Unpaired two-tailed Student's *t*-test was used for the comparison of the two groups. ns, not significant.

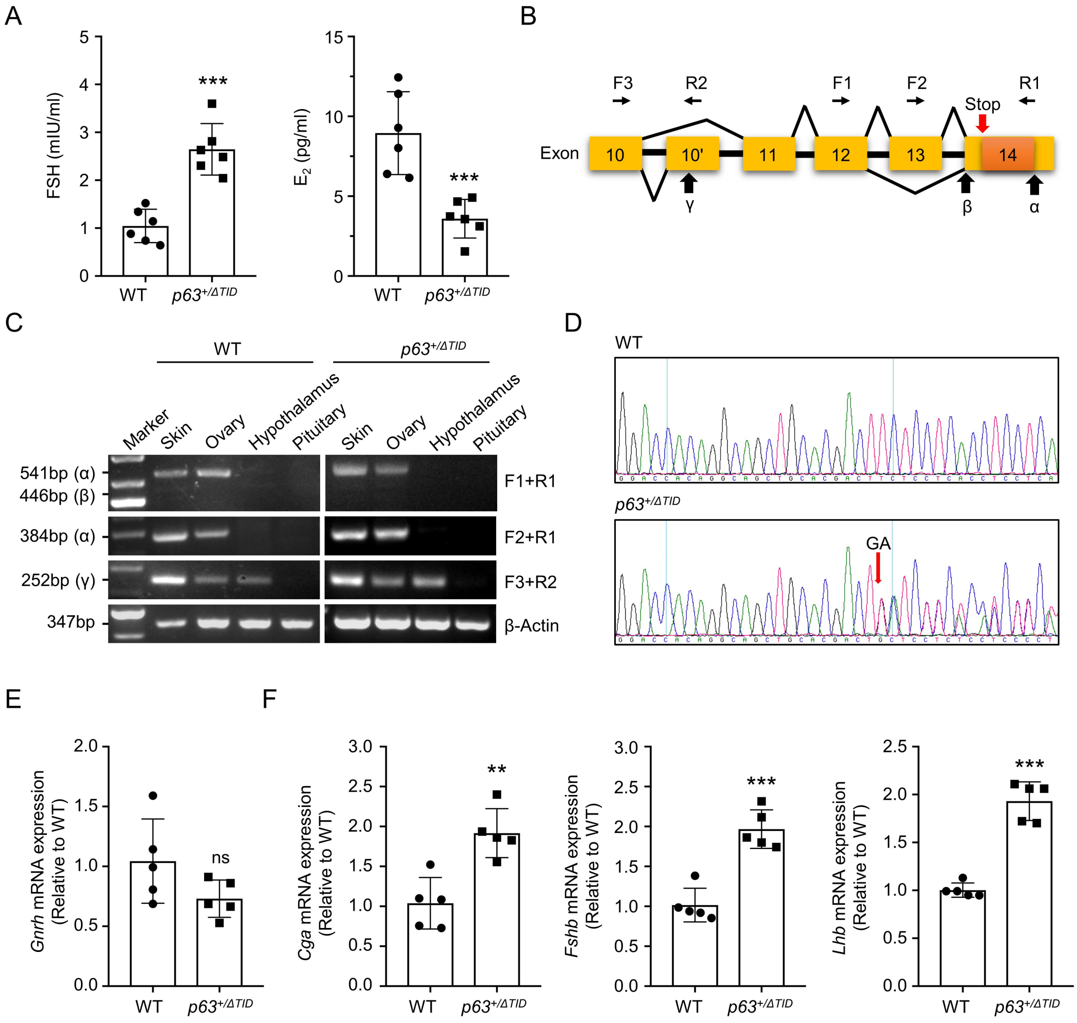

Supplemental Figure 4. Expression of *p63* and functional analysis of the hypothalamus and pituitary in *p63*$^{+/\Delta TID}$ mice. (A) Serum follicle-stimulating hormone (FSH) and Estradiol (E_2) levels were measured in 2M WT and *p63*$^{+/\Delta TID}$ mice (n = 6 for each genotype). **(B)** C-terminal structure of the *p63* gene. The primers F1, F2, F3, R1, and R2 are indicted by horizontal black arrows. The stop codons of *p63α*, *p63β*, and *p63γ* are indicted by vertical black arrows, and the introduced stop codon in *p63α* is indicated by the red arrow. **(C)** Semi-quantitative RT-PCR analysis of *p63* isoform expression in the P1 epidermis, P1 ovary, 2M hypothalamus, and 2M pituitary of WT and *p63*$^{+/\Delta TID}$ mice. The three isoforms amplified by the primers are indicated in parentheses. The *p63β* isoform with a predicted band size of 446 bp was not detected. **(D)** Sanger sequencing of the PCR products of F1+R1 primers obtained by amplifying the cDNA from P1 WT and *p63*$^{+/\Delta TID}$ ovaries. The red arrow indicates the site of GA insertion. **(E and F)** Quantitative RT-PCR analyses of the *Gnrh* gene in

the hypothalamus and the *Cga, Fshb*, and *Lhb* genes in the pituitary of 2M WT and *p63*$^{+/\Delta TID}$ mice. *Gapdh* was used as the internal control. n = 5 for each phenotype. In panel A, E, and F, data are presented as the mean ± SD, and differences between the groups were analyzed for statistical significance by the unpaired two-tailed Student's *t*-test. $^{**}P < 0.01$, $^{***}P < 0.001$, ns, not significant.

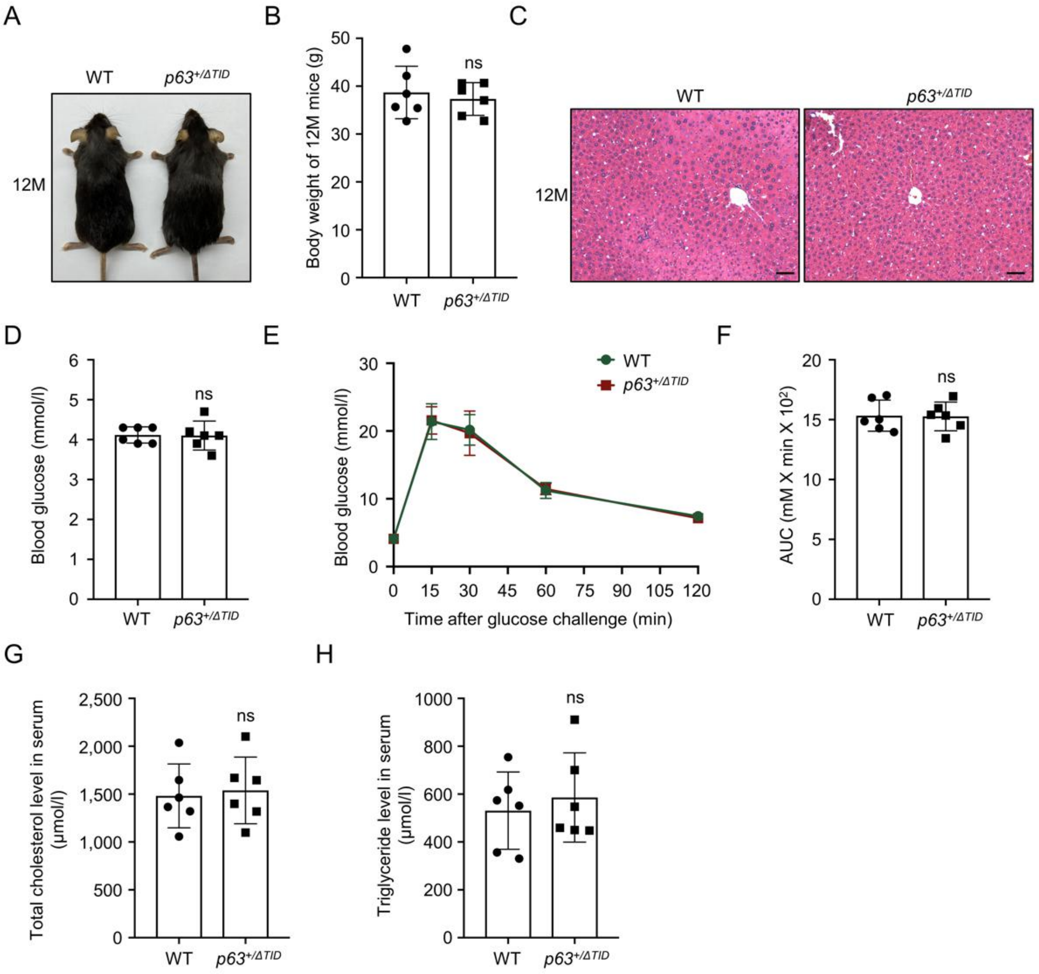

Supplemental Figure 5. Metabolic phenotypes of WT and *p63*$^{+/\Delta TID}$ mice. (A) Gross morphology of 12M WT and *p63*$^{+/\Delta TID}$ females. **(B)** No significant differences were observed in body weight between 12M WT and *p63*$^{+/\Delta TID}$ mice. n = 6 per group. **(C)** HE staining of liver sections of 12M WT and *p63*$^{+/\Delta TID}$ mice. Scale bar: 50 μM. **(D and E)** Fasting blood glucose levels and results of the glucose tolerance test performed in 2M WT and *p63*$^{+/\Delta TID}$ mice fasted for 16 h. Each point on the graph indicates the level of glucose in the blood. Blood glucose levels were detected at 15 min, 30 min, and 60 min in the glucose tolerance test. n = 6 per group. **(F)** Areas under the curves (AUC) calculated from mice in the glucose tolerance test. n = 6 for each genotype. **(G and H)** Serum total cholesterol and triglyceride levels in 2M WT and *p63*$^{+/\Delta TID}$ mice. n = 6 for each genotype. In panel B, D, and F-H, data are presented as the mean ± SD, and differences between the groups were analyzed for statistical significance by the unpaired two-tailed Student's *t*-test. ns, not significant.

Supplemental Table 1. Clinical characteristics of POI patients with mutations in *TP63*.

Patient ID	Phenotype	Menarche age (yr)	Amenorrhea age (yr)	Age at diagnosis (yr)	FSH (IU/l)	E_2 (pg/ml)	Left ovary (cm*cm)	Right ovary (cm*cm)
1	SA	13	16	28	88.88	20	Invisible	Invisible
2	SA	15	29	38	88.27	<5	1.5*0.7	1.6*1
3	SA	13	19	28	67.3	<5	1.4*0.7	1.4*1
4	PA	-	-	32	34.51	11.5	Invisible	1.3*0.5
5	PA	-	-	27	48.73	<5	Invisible	Invisible
6	PA	-	-	24	54.6	<5	1.1*0.5	1.4*0.7
7	PA	-	-	23	70.91	<5	Invisible	Invisible
8, 9, 10	SA	13; 19; 16	25; 19; 16	27; 28; 30	55.77; 78.32; 26.45	<5; 20; <5	1.5*0.8; Invisible; 1.4*1.1	1.4*0.6; Invisible; 1.7*0.8
11	SA	13	13	28	67.16	<5	1.7*0.7	1.7*0.6

E_2, estradiol; FSH, follicle-stimulating hormone; POI, premature ovarian insufficiency; PA, primary amenorrhea; SA, secondary amenorrhea; yr, years; -, not available.

Supplemental Table 2. Comparison of amino acid sequences in different C-terminal knockout strategies.

Name	Sequences	Auto-activation in oocyte	Reference
Cα	VRGRETYEMLLKIKESLELMQYLPQHTIETYRQQQQQHQHLLQKQTSMQSQ SSYGNSSPPLNKMNSMNKLPSVSQLINPQQRNALTPTTMPEGMGANIPMMGT HMPMAGDMNGLSPTQALPPPLSMPSTSHCTPPPPYPTDCSIVSFLARLGCSSCL DYFTTQGLTTIYQIEHYSMDDLASLKIPEQFRHAIWKGILDHRQLHDFSSPPHLL RTPSGASTVSVGSSETRGERVIDAVRFTLRQTISFPPRDEWNDFNFDMDSRRNK QQRIKEEGE*	-	Lena AM, et al. Nat Commun, 2021; Suh EK, et al. Nature, 2006
CαΔTID	VRGRETYEMLLKIKESLELMQYLPQHTIETYRQQQQQHQHLLQKQTSMQSQ SSYGNSSPPLNKMNSMNKLPSVSQLINPQQRNALTPTTMPEGMGANIPMMGT HMPMAGDMNGLSPTQALPPPLSMPSTSHCTPPPPYPTDCSIVSFLARLGCSSCL DYFTTQGLTTIYQIEHYSMDDLASLKIPEQFRHAIWKGILDHRQLHD*	+	This study
Cβ	VRGRETYEMLLKIKESLELMQYLPQHTIETYRQQQQQHQHLLQKQTSMQSQ SSYGNSSPPLNKMNSMNKLPSVSQLINPQQRNALTPTTMPEGMGANIPMMGT HMPMAGDMNGLSPTQALPPPLSMPSTSHCTPPPPYPTDCSIVRIWQV*	+	Lena AM, et al. Nat Commun, 2021
Cα'	VRGRETYEMLLKIKESLELMQYLPQHTIETYRQQQQQHQHLLQKQTSMQSQ SSYGNSSPPLNKMNSMNKLPSVSQLINPQQRNALTPTTIPDGMGANIS*	-	Suzuki D, et al. Development, 2015
Cγ	VRGRETYEMLLKIKESLELMQYLPQHTIETYRQQQQQHQHLLQKHLLSACF RNELVEPRGEAPTQSDVFFRHSNPPNHSVYP*	-	Lena AM, et al. Nat Commun, 2021

Green, SAM domain; Red, TID domain; Blue, essential for activation in oocytes (EAO) domain; *, stop codon; Underline, sequences of Cβ and Cγ different from Cα; +/-, auto-activation/inactivation in oocyte.

Supplemental Table 3. Primers used in this study.

Primer	Sequence (5'-3')	Product
**Genotyping *p63*$^{+/\Delta TID}$ mice**		
Forward	CATTTAAGCCAAAACACCAGAGAGT	387bp
Reverse	ATTCTCCTTCCTCTTTGATACGCT	
**Genotyping *p63*$^{+/R647C}$ mice**		
Forward	TGAGGCCAGTGGAGAACAAG	582bp
Reverse	GCCTCCTAATTCTCCGTCCC	
**qPCR**		
Noxa-Forward	GTTCGCAGCTCAACTCAGGA	72bp
Noxa-Reverse	ACCACAGTTATGTCCGGTGC	
Puma-Forward	CAGCACTTAGAGTCGCCCG	196bp
Puma-Reverse	GTGAGGGTCGGTGTCGATG	
Gapdh-Forward	AGGTCGGTGTGAACGGATTTG	123bp
Gapdh-Reverse	TGTAGACCATGTAGTTGAGGTCA	
Gnrh-Forward	GGGAAAGAGAAACACTGAACA	98bp
Cnrh-Reverse	GGACAGTACATTCGAAGTGCT	
Cga-Forward	CTGTTGCTTCTCCAGGGCATA	66bp
Cga-Reverse	TTCTTTGGAACCAGCATTGTCTT	
Fshb -Forward	GGAGAGCAATCTGCTGCCATA	77bp
Fshb-Reverse	GCAGAAACGGCACTCTTCCT	
Lhb -Forward	TGGCCGCAGAGAATGAGTTC	84bp
Lhb-Reverse	CTCGGACCATGCTAGGACAGTAG	
**Semi-quantitative RT-PCR**		
F1-Forward	GACTCAGCCCTACCCAAGCTCTC	F1+R1 (α/β):
R1-Reverse	TGTAGGGGCTGGGAGGTGGAAG	541bp/446bp
F2-Forward	GACCACCATCTATCAGATTGAGC	F2+R1 (α):
R1-Reverse	TGTAGGGGCTGGGAGGTGGAAG	384bp
F3-Forward	GTGAGAGGTCGTGAGACGTAC	F3+R2 (γ):

R2-Reverse	CTATGGGTACACGGAGTGGTT	252bp
β-Actin-Forward	TGTCCCTGTATGCCTCTGGTCG	347bp
β-Actin-Reverse	GAACCGCTCGTTGCCAATAGTG	
**Human mutations**		
Forward (c.854)	TTTTGCCACCAACATCCTGT	387bp
Reverse (c.854)	CTGAAGGGAAAGCATGTGGAG	
Forward (c.1612)	TGTAGGCTGTTTGAAGGGGT	653bp
Reverse (c.1612)	GAGTAAGTGAAGGCGAGGGA	
Forward (c.1700, 1703)	TGTCACCAGTAATCTCCAGACC	543bp
Reverse (c.1700, 1703)	CTCCTCTTTCCCACCTTGAGA	
Forward (c.1780, 1928, 1937, 1939, 1964)	GTTCTACACAGGCAGGAAAGAC	697bp
Reverse (c.1780, 1928, 1937, 1939, 1964)	GCCAGAATCAGAATTAGATGCCA	
